# The COVID-19 Pandemic in Romania: A Comparative Description with Its Border Countries

**DOI:** 10.3390/healthcare10071223

**Published:** 2022-06-30

**Authors:** Bianca Georgiana Enciu, Alina Andreea Tănase, Anca Cristina Drăgănescu, Victoria Aramă, Daniela Pițigoi, Maria-Dorina Crăciun

**Affiliations:** 1 Department of Epidemiology, Carol Davila University of Medicine and Pharmacy, 020021 Bucharest, Romania; bianca.milcu@drd.umfcd.ro (B.G.E.); maria.craciun@umfcd.ro (M.-D.C.); 2 National Centre for Communicable Diseases Surveillance and Control, National Institute of Public Health, 050463 Bucharest, Romania; 3 University Emergency Hospital from Bucharest, 050098 Bucharest, Romania; alina-andreea.tanase@rez.umfcd.ro; 4 Department of Pediatrics, Carol Davila University of Medicine and Pharmacy, 020021 Bucharest, Romania; drdraganescu@yahoo.com; 5 National Institute for Infectious Diseases “Prof. Dr. Matei Balș”, 021105 Bucharest, Romania; victoria.arama@umfcd.ro; 6 Department of Infectious Diseases, Carol Davila University of Medicine and Pharmacy, 020021 Bucharest, Romania; 7 Department of Infection Prevention and Control, Emergency Clinical Hospital for Children “Grigore Alexandrescu”, 011743 Bucharest, Romania

**Keywords:** COVID-19 pandemic, vaccines, Romania, border, characteristics

## Abstract

The COVID-19 pandemic continues to be a major public health problem in most countries of the world, especially in developing countries with an underfunded healthcare system. We aim to present a comparative profile of the epidemiological characteristics of the COVID-19 pandemic in Romania and neighboring countries, which have similar onset and a similar socio-cultural pattern. A descriptive comparative study was performed using COVID-19 data collected from various official websites regarding demography, morbidity, mortality, vaccination, and testing capacity. The countries included in the study were Romania, Bulgaria, Hungary, Republic of Moldova, Serbia, and Ukraine. The study period was from week 09/2020 to week 46/2021. Overall, these countries have reported 8,382,441 cases and 216,014 deaths (during the study period). The highest cumulative incidence rate of cases has been recorded in Serbia (17,801.5) and the highest mortality rate has been recorded in Bulgaria (391.0). Romania is in fourth place regarding the cumulative incidence rate of cases/100,000 inhabitants but in third place regarding the mortality due to COVID-19 (case–fatality rate of 3.1%). Although the World Health Organization and EU co-ordinate the COVID-19 response, each state makes its own decisions regarding SARS-CoV-2 mitigation measures, the epidemiological indicators directing us about the effectiveness of responses.

## 1. Introduction

SARS-CoV2 virus has spread rapidly around the world, causing high morbidity and mortality. Since December 2019, when the first cases of pneumonia of unknown etiology were reported in Wuhan, China, the numbers of reported infections and deaths have increased significantly [1,2,3].

The speed with which the first measures were implemented, the level of awareness of the danger, the ability to protect medical staff and to perform contact tracing, subsequent access to vaccines, and public confidence in them have been decisive factors in reducing the spread of the pandemic [3]. However, the effectiveness of responses varies from country to country [3,4].

In Europe, the European Union (EU) co-ordinates the COVID-19 response in the Member States and provides the financial and logistical resources needed to produce, purchase, and distribute COVID-19 vaccines [5,6]. Nevertheless, governments are taking individual decisions on measures to reduce the spread of the virus [4,6].

In Romania, the authorities follow the World Health Organization (WHO) recommendations, implementing measures similar to those implemented in other countries affected by COVID-19. However, Romania faced several challenges in the various stages of the pandemic, including the large number of Romanian citizens returning from the already affected areas, inadequate infrastructure of the healthcare system, and socio-cultural determinants [3,6,7,8]. A part of these challenges was described in papers already published, but there are few studies comparing the evolution of the pandemic in this area of Europe from the perspective of control measures. A comparative analysis of the epidemiological indicators between Romania and its border countries could bring a better understanding of the public health implications of the COVID-19 pandemic in this area. These countries, hosting about 13% of the European population, had similar onset of the COVID-19 pandemic and a similar socio-cultural pattern. The aim of this study is to present a comparative profile of the epidemiological characteristics of the COVID-19 pandemic in Romania and neighboring countries in order to draw recommendations for improving the response to the current pandemic and to prepare for possible similar future situations.

## 2. Materials and Methods

We performed a retrospective comparative study of the main statistical indicators describing the evolution of the COVID-19 pandemic data and the control measures adopted in Romania (RO) and the neighboring countries: Bulgaria (BG), Hungary (HUN), Republic of Moldova (MDA), Serbia (SRB), and Ukraine (UKR). The following indicators were compared: morbidity, mortality, vaccination, and testing capacity. Statistical data were collected from official sources, such as United Nation website [9], World Bank website [10], World Health Organization website [11], Our World in Data managed by Oxford University [12], and European Centre for Disease Control websites [13,14]. The study period was between week 09/2020 and week 46/2021.

The Microsoft Excel program was used to collect and analyze data. Weekly cumulative cases and deaths were used to describe the time trend.

## 3. Results

From all these six countries included in the study, three of them are Member States of the European Union (Romania, Bulgaria, and Hungary).

The main demographic and economic indicators of the included countries are presented in Table 1.

**Figure 1:**
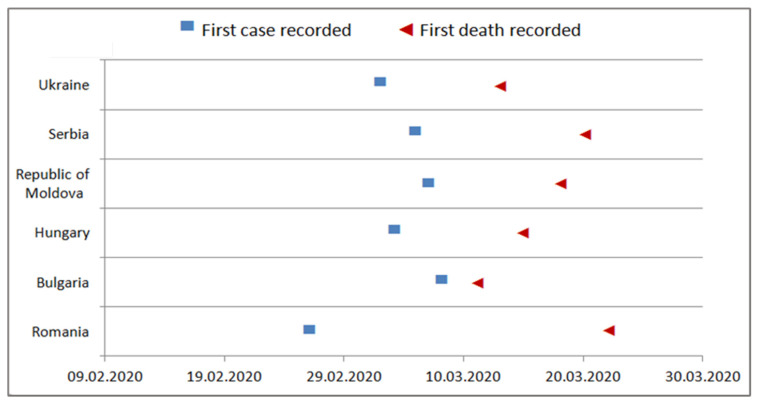
The onset of the COVID-19 pandemic by country [11,12,13].

The onset of the COVID-19 pandemic in the six countries included in the study occurred at the same time, the end of February–the beginning of March 2020 (weeks 9–11/2020) (Figure 1).

During the studied period, the included countries have reported 8,382,441 cases, representing 10.3% of reported cases in Europe (Europe = 81,540,105). Ukraine has reported the highest number of cases (3,340,407), followed by Romania (1,752,442), but the highest cumulative incidence rate per 100,000 inhabitants has been recorded in Serbia (17,801.5). In total, 216,014 deaths have been reported during the study period, representing 14.5% of reported deaths in Europe (Europe = 1,491,599).

The highest number of deaths has been recorded in Ukraine (81,598), followed by Romania (55,113), but the highest mortality rate has been recorded in Bulgaria (391.0) (Table 2).

Since the beginning of the pandemic until week 46/2021, the countries included in the study have been affected by three pandemic waves, the breadth of each pandemic wave varying by country.

**Figure 2:**
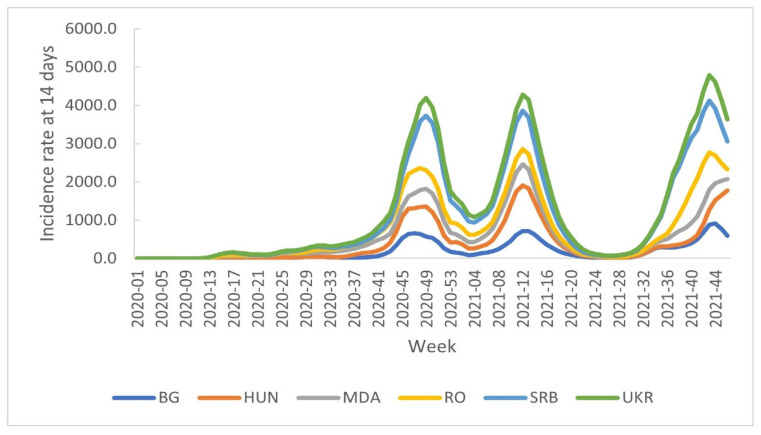
The distribution of cumulative incidence rate of cases at 14 days by country, week 9/2020-week 46/2021.

After the first two pandemic waves from the end of 2020 and the beginning of 2021, respectively, the intensity of the pandemic in all studied countries has begun to progressively decrease until week 25/2021, when a subtle increase in cumulative incidence rate of cases at 14 days was recorded in the Republic of Moldova. The same trend became noticeable in all countries after week 28/2021 (Figure 2).

**Figure 3:**
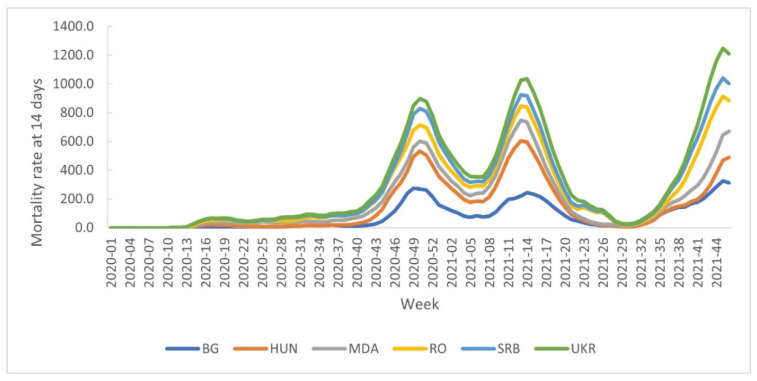
The distribution of mortality rate at 14 days by country, week 9/2020-week 46/2021.

A high increase in the number of COVID-19 cases was recorded from week 32/2021, Romania and Serbia recording more cases and deaths than those recorded during previous waves (Figure 2 and Figure 3).

Most tests were performed in Romania (15,916,982) but reported to 100 inhabitants, we will notice that Serbia is in first place, with a testing rate of 97.6%, followed by Bulgaria (92.5%). Ukraine (34.6%) has performed fewer COVID-19 tests/100 inhabitants. Regarding the average number of tests performed per case, the highest value was calculated for Bulgaria (10 tests/case). Lower values were reported in Serbia and Ukraine (5 tests/case) (Table 3).

The vaccination campaigns started at the end of December 2020 in all the three EU Member States and in Serbia. In the Ukraine and the Republic of Moldova, the access to the vaccines was delayed to the beginning of February and March 2021, respectively. During the study period, Romania and Bulgaria used only the four vaccines authorized by the European Medicines Agency.

Since the beginning of the vaccination campaigns until week 46/2021, 60,728,342 doses of COVID-19 vaccines were administered in the studied countries, 25,808,707 people being fully vaccinated and 32,554,160 receiving at least one dose (Table 4).

Hungary is placed on the top position, having more than 50% of the population fully vaccinated against COVID-19. At the opposite pole is the Republic of Moldova, where 15.6% of inhabitants are fully vaccinated (Table 4).

## 4. Discussion

The COVID-19 pandemic continues to represent a major public health problem in most countries of the world, especially in developing countries, with an underfunded healthcare system and a low degree of public confidence in the authorities. Member States of community blocks, such as the European Union, have received support in managing the COVID-19 pandemic [4,6]. The governments’ response, the behavior of the population, as well as the solidarity between states are decisive factors in shaping the epidemiological characteristics of each country [5]. There are differences in the epidemiological indicators from each country, despite cultural, economic, or social similarities [7].

In this paper, we set out to present the characteristics of the COVID-19 pandemic in Romania compared to neighboring states. Romania shares with these countries not only borders, but also some common features.

The Romanian authorities’ response was prompt, despite the challenges faced at the beginning of the pandemic (increased flow of Romanian citizens returning from countries affected by the pandemic, lack of medical equipment, and insufficient medical staff) [3,6].

Romania, compared to the other states included in this research, recorded a lower incidence rate during the study period, but this parameter depends on the testing capacity, which has evolved progressively since the beginning of the pandemic in all studied countries.

Like most of the countries included in the study, excepting Serbia, Romania has reported a high case–fatality rate, superior to the European average. The increased case fatality rate may indicate a difficulty of the healthcare system in managing severe cases but, for a relevant opinion, it is necessary to consider other issues, such as the profile of deceased people (age and comorbidities), type of transmission in the population (community transmission and clusters), location of cases, and level of testing (early diagnosis). A particular situation was noticed in Serbia, a non-EU member state having a social dynamic similar to Romania. Here, we observed a high testing rate with a high incidence rate, but lower mortality and fatality rates, below the European average [8,15,16]. This situation highlights the importance of testing for early detection and treatment of cases in order to reduce the severity and the case fatality rate. Romania and other countries included in the study should follow the example of Serbia and increase the testing capacity.

The cumulative incidence rate at 14 days was one of the indicators that guided the pandemic management measures in Romania, but decisions should not be implemented only based on this parameter. Other factors should be taken into consideration, such as the evolution of the vaccination campaign, the severity of cases, and the circulation into the community of new SARS-CoV-2 strains [17].

Romania, like the other countries included in the study, faced three pandemic waves during the study period, by comparison with other European countries, which were hit by four pandemic waves. The intensity of each wave depended on the virulence of the SARS-CoV-2 circulating strain, the level of preparedness of the healthcare system to treat severe cases, the mitigation measures implemented by the authorities, the compliance of the population to the authorities’ recommendations, and the evolution of the vaccination campaign.

Romania and Bulgaria are placed on the last positions regarding vaccination uptake among EU countries, despite the early access to the COVID-19 vaccines and a good onset of the vaccination campaign [5]. By comparison, non-EU Member States, excepting Serbia, have difficulty in accessing COVID-19 vaccines, this situation leading to a low vaccination coverage. In order to have a high vaccination coverage, which reduces the spread of SARS-CoV-2 virus, it is important to know the main factors conducting to a low vaccine uptake. In Romania, vaccine hesitancy represents the main factor leading to a low vaccine uptake, suggested also by the unsuccessful HPV vaccination campaign from 2008 and by the measles epidemic started in 2016 determined by the low MMR vaccination coverage. The situation is similar in Bulgaria.

In the Republic of Moldova and Ukraine, the main factor involved in low vaccination coverage is the difficulty of accessing vaccines.

Therefore, it is important to identify the causes for vaccine hesitancy in Romania and Bulgaria and to ensure equal access to vaccines in all countries.

One issue of the citizens’ low compliance with the measures implemented is their trust level in their governments and healthcare system. In order to control the spread of the disease, people’s compliance with the containment measures should be ensured on the long term; this can be achieved using awareness campaigns about COVID-19 burden, by persuading citizens about the effectiveness of the proposed measures, providing information about vaccines’ efficacy and safety, and also about their adverse events.

The Romanians’ interest for vaccination has been influenced by the containment measures implemented. For example, after lifting some COVID-19 containment measures (on 1 May 2021) the interest in vaccination decreased. Moreover, in October 2021, when European COVID-19 Digital Certificate became compulsory for entering some public places, the interest increased.

Increasing the uptake of COVID-19 vaccine among eligible individuals, raising awareness about the importance of the mitigation measures together with the early diagnosis and treatment of COVID-19 cases are the factors that will decisively influence the evolution of the COVID-19 pandemic in all studied countries, including Romania. Moreover, taking into consideration the low vaccination coverage from Ukraine and the higher number of refugees coming from this country, it is important that every state hosting these people ensure the COVID-19 vaccine to those eligible who do not own a vaccination proof.

General recommendations to all countries in this context are: raising awareness about the importance of the vaccination and containment measures and about the COVID-19 burden, increase the testing capacity to reduce the fatality of the disease, create a legislative framework to sustain a higher vaccination uptake, ensure the COVID-19 vaccine for all unvaccinated refugees, and use boosters to counteract decrease in immunity.

Policy implications: we consider it necessary that all countries included in the study should have in place a legislative framework sustaining vaccination (including communication and educational campaigns, stakeholders involved and their attribution, vaccine acquisition and adequate stocks, etc.) and an epidemiological emergency preparedness plan, so, in the eventuality of other similar events, the response will be quicker and more adequate.

All countries should learn from each other, as well as from other countries. We should always remember that a virus or bacteria does not respect any border, so it is necessary to co-operate and to help each other, no matter the membership, for the common wellbeing.

This study has a set of strengths, such as the fact that it is the first study providing a comparative analysis between Romania and its border countries and offering some public health recommendations for managing this current pandemic but also for other similar situations.

Limitations of this study are related to the lack of homogenous data regarding the COVID-19 epidemiological indicators, especially from non-EU countries. Moreover, we had not enough information about the characteristics of cases and deaths (age and co-morbidities) and about the number of antigenic tests performed at home.

Another limitation is related to the lack of data regarding the mitigation measures implemented in all countries included in the study. We have found data regarding only EU Member States.

A comparison into a broader context, including all European countries, could provide a better picture but we assume the limitation of studying only neighbouring countries to Romania.

## 5. Conclusions

In conclusion, this paper underlines the main similarities and differences in the response of the authorities and population to the COVID-19 pandemic from Romania and its neighboring countries.

In the study period, the incidence rate was below the European average (all European countries, not only EU countries). The mortality rate and the case fatality rate were above the European average.

Romania had an average position in the region regarding cumulative incidence rate, below Serbia, Hungary, and Bulgaria and above the Republic of Moldova and Ukraine, as well as regarding cumulative mortality rate, below Bulgaria and Hungary and above the Republic of Moldova, Ukraine, and Serbia.

Regarding vaccine coverage, the region was much below the EU average. At the country level, Romania was below Hungary and Serbia and above Ukraine, Bulgaria, and Moldova.

## Figures and Tables

**Table 1:** The number of inhabitants and income level, by country [9,10].

Country	Population (Million)	GDP per Capita (USD)	Economic Level
Romania	19.3	12,915	Upper income country
Bulgaria	7.0	10,079	Upper middle-income country
Hungary	9.8	15,980	Upper income country
Republic of Moldova	4.0	4547	Lower middle-income country
Serbia	6.9	7731	Upper middle-income country
Ukraine	43.7	3725	Lower middle-income country

**Table 2:** The COVID-19 pandemic characteristics by country (until week 46/2021) [11,12,13].

Country	Romania	Bulgaria	Hungary	Republic of Moldova	Serbia	Ukraine
**Cumulative number of cases**	152,442	672,555	1,025,778	358,202	1,233,057	3,340,407
**Cumulative incidence rate (per 100,000 inhabitants)**	9066.5	9675.0	10,499.8	8879.7	17,801.5	7638.1
**Cumulative number of deaths**	55,113	27,180	32,064	8834	11,225	81,598
**Cumulative deaths rate** **(per 100,000 inhabitants)**	285.1	391.0	328.2	219.0	162.1	186.6
**Case fatality rate (%)**	3.1	4.0	3.1	2.5	0.9	2.4

**Table 3:** COVID-19 testing by country, week 9/2020-week 46/2021 [11].

Country	Total Tests	Number of Tests/100 Inhabitants	Number of Tests/Case
Bulgaria	6,433,278	92.5	10
Hungary	7,847,984	80.3	8
Republic of Moldova	2,101,912	52.1	6
Romania	15,916,982	82.3	9
Serbia	6,759,262	97.6	5
Ukraine	15,128,821	34.6	5

**Table 4:** The vaccination situation by country (since the beginning of the vaccination campaign in each country until week 46/2021) [11].

Country	Romania	Bulgaria	Hungary	Republic of Moldova	Serbia	Ukraine
**Total number of doses** **Administered**	13,577,218	3,189,047	13,369,134	1,592,367	6,353,068	22,647,508
**Fully vaccinated**	5,441,352	1,295,027	5,573,306	628,833	3,096,056	9,774,133
**At least one dose**	7,657,261	1,832,182	5,970,796	963,534	3,257,012	12,873,375
**Total number of doses** **administered/100 inhabitants**	70.2	45.9	136.8	39.5	91.7	51.8
**Fully vaccinated/100** **Inhabitants**	28.2	18.6	57.9	15.6	44.7	22.3
**At least one dose/100** **Inhabitants**	39.6	26.4	61.1	23.9	47.0	29.4

## Data Availability

The datasets generated and analyzed during the current study are available from the corresponding author upon reasonable request.
